# Laparoscopic Gynecology Procedures: Avoid the Risk

**DOI:** 10.1155/DTE.2.157

**Published:** 1996

**Authors:** James E. Carter

**Affiliations:** 1 Advanced Surgical Education Associates Mission Viejo California USA; 2 F.A.C.D.G. Woman's Health Center 26732 Crown Valley #541 Mission Viejo CA 92691 USA

## Abstract

Laparoscopic approaches to gynecological surgery have been developed by an elite group of highly skilled surgeons. As these procedures become more prevalent in the general gynecological approach to disease and the general gynecologist's approach to treatment, the complication rate for these procedures is likely to increase. In an effort to assist in avoiding these complications, guidelines for the performance of laparoscopic gynecological procedures need to be established. This article presents approaches to the most common gynecological procedures that can assist in the prevention of complications.


*Diagnostic and Therapeutic Endoscopy*, 1996, Vol. 2, pp. 157–166
Reprints available directly from the publisher
Photocopying permitted by license only

© 1996 OPA (Overseas Publishers Association)
Amsterdam B.V. Published in The Netherlands
by Harwood Academic Publishers GmbH
Printed in Singapore

### Laparoscopic Gynecology Procedures: Avoid the Risk

JAMES E. CARTER

*Advanced Surgical Education Associates, Mission Viejo, California*

*(Received June 2, 1995; in final form August 4, 1995)*

Laparoscopic approaches to gynecological surgery have been developed by an elite group of highly skilled surgeons. As these procedures become more prevalent in the general gynecological approach to disease and the general gynecologist's approach to treatment, the complication rate for these procedures is likely to increase. In an effort to assist in avoiding these complications, guidelines for the performance of laparoscopic gynecological procedures need to be established. This article presents approaches to the most common gynecological procedures that can assist in the prevention of complications.

**KEY WORDS:** Laparoscopy, complications, bowel injury, Richter's hernia

### INTRODUCTION

A short review of the literature on the laparoscopic hysterectomy (1–19) revealed the following complications: bladder injuries including lacerations, punctures, vesicle-vaginal fistulas; ureteral injuries including ureterovesicle fistulas, ureteral transections, and cauterizations; bowel injuries including enterotomies, thermal injuries, and herniations into trocar sites; nerve injuries; hemorrhage both intraoperative and postoperative, some requiring transfusion; injuries to abdominal wall vessels and retroperitoneal vessels; infections including pelvic abscesses requiring drainage, cellulitis, and pneumonia, with one postoperative case of pneumonia resulting in death; anesthetic problems including cardiac arrythmias and pulmonary edema; and vascular problems including deep venous thrombosis and pulmonary embolus.

Of all the laparoscopic gynecological procedures, only the complication rate of the laparoscopic-assisted vaginal hysterectomy (LAVH) and laparoscopic hysterectomy has been studied and recorded. In one such study by Padial et al. (13) patients undergoing LAVH had a 10.6% frequency of febrile morbidity and no organ injuries or wound infections. Those having a vaginal hysterectomy during the same time frame experienced a 31% rate of febrile morbidity, with one instance each of a bladder and a bowel injury, and no wound infections. The women treated by abdominal hysterectomy experienced a 52% rate of febrile morbidity, with two instances each of bowel and bladder injury, two blood transfusions, and four wound infections.

As our procedures improve and our surgical skills mature, the already low instance of complications with the laparoscopic approach to gynecological procedures can be expected to fall further. The purpose of this article is to provide the laparoscopic gynecological surgeon with techniques to minimize even further the complication rate. This article addresses the following areas: patient preparation; anesthesia considerations; patient position; insufflation and trocar placement; treatment of vascular pedicles; minimizing the risk of bladder, bowel, and ureter injury; minimizing the risk of infection; and minimizing the risk of thromboembolic events.

### PATIENT PREPARATION

Patient preparation should include careful counseling of the patients for the possibility of an open procedure as well as counseling on all of the risks of laparoscopic surgery (20–27).

The patient should be instructed for all advanced laparoscopic procedures and undergo a bowel cleansing procedure with the use of an osmotic agent such as GoLITELY (25,28). Prior to initiation of the procedure the patient

Address for correspondence: James E. Carter, M.D., Ph.D., F.A.C.D.G., Woman's Health Center, 26732 Crown Valley #541, Mission Viejo, CA 92691.

should be given intravenous antibiotics such as cefotam (1 g) and metronidazole (500 mg) for infection prophylaxis.

Before using this protocol, the author experienced two vaginal cuff infections after LAVH. Since this protocol has been used, there have been no vaginal cuff infections in 200 LAVHs.

The anesthesiologist should be instructed to place a nasogastric or orogastric tube for decompression of the stomach and should be instructed to avoid the use of nitrous oxide. A Foley catheter should be placed to empty the bladder. In addition, the patient should not be placed in the Trendelenberg position until the umbilical trocar has been placed (29,30). Fluid overload should be avoided as the process of operating under 15 mm Hg intra-abdominal pressure and a steep Trendelenberg position will reduce urine output, in spite of good hydration. The fluids should be warmed and the patient maintained at normal temperature throughout the procedure (3). The anesthesiologist should maintain vigilance for any activity that would indicate carbon dioxide embolism and should monitor the patient carefully for hypercapnea (32–34).

The patient should be positioned with the arms next to the sides to provide the surgeon with adequate working space and to avoid the possibility of injury from a hyperextended position at the shoulder. A nonskid pad should be placed under the patient, who should be placed on a nonelectric table that can provide sufficient Trendelenberg position for the surgical procedures to be performed. Regardless of the stirrup systems that will be used, great care must be exercised to ensure that no pressure is placed on nerve structures, especially the peroneal nerve. The legs should be carefully positioned so that excess stretch of nerves such as the femoral or sciatic nerves is avoided (3). Pneumatic compression boots should be placed on the lower extremities and activated during the surgical procedure.

### INSUFFLATION AND TROCAR PLACEMENT

### Veress Needle Technique

Placement of the initial Verres needle puncture, direct trocar puncture, or opening of the incision (if an open laparoscopy is performed) should only occur after the surgeon has carefully cleaned the umbilical site for any remnant of contamination. This site is extremely difficult to clean and frequently must be approached with the use of a Kelly forceps to extract all material. Cellulitis can be avoided if this site is meticulously cleaned. The umbilical site is the preferred site for initial placement of either the Veress needle, direct puncture, or the open Hasson procedure for the first incision by most laparoscopic gynecologists (2,36–38).

This site, however, has three major disadvantages. First, the transverse colon will frequently lay directly under or even caudad to the umbilical site. The second disadvantage is that the mesentery of the bowel also frequently lies directly under the umbilical site. Finally, the aorta, vena cava, and especially the left common iliac vein are frequently in close proximity to, directly below, or caudad to the umbilical site. Therefore, when the intraumbilical incision is made (which is preferable to a subumbilical incision because of the direction of the lines of Langham at the umbilical site) the Veress needle must be inserted only millimeters through the fascia, which is elevated under tension if the Veress needle is inserted in a vertical direction. If the Veress needle is inserted in a caudad direction, the skin should be elevated and the Veress needle inserted in a direction toward the uterus in the deep pelvis. This technique will traverse fat, fascia, and peritoneum before entry.

If the Veress needle technique is used, the position of the Veress needle must be confimed by injection of 20 ml of normal saline and withdrawing the syringe. Any evidence of fluid return should be interpreted based on the color and odor of the fluid. Fluid return that is clear indicates that the Veress needle has not properly punctured the peritoneum. The Veress needle should be withdrawn and replaced. Fluid that is red in color indicates the possibility of a vascular injury and must be interpreted based on the color and amount of fluid. Fluid that is brown and has an odor obviously signifies puncture into the bowel, and the Veress needle in this case can be left in position and an optional trocar placement be performed so that the position of the puncture can be identified. An alternate approach for this would be simply to remove the Veress needle, appreciating the fact that frequently the bowel is very likely entered by the needle and, because of its size, is not subject to great injury as long as the needle is not moved randomly to increase the amount of the injury. This author prefers to leave the Veress needle in place and evaluate the injured site if this occurs. The Veress needle can also enter the mesentery and create an emphysematous reaction in the mesentery of the bowel. The proper reaction after injection of the 20 ml of normal saline is to feel suction pressure against the syringe, indicating proper placement of the Veress needle.

The gas supply is then attached. It is advised to use a large bore tubing for placement of the gas as the resistance to flow of gas in a tube is inversely proportional to the fourth power of the radius of the tube. Doubling the radius of the tube reduces the resistance to flow of the gas by a factor of 16. The pressure of insufflation should be monitored early in the introduction of the gas supply. If the Veress needle is properly positioned, the pressure noted on the gauge will be less than 6 mm Hg. If the pressure is >6 mm but <10 mm, it is possible that the Veress needle has found its way into the mesentery or into the bowel. If the pressure is >10 mm, it is very possible that the Veress needle is, in fact, not in the peritoneal cavity. Flow rate at insufflation should be maintained at 1 liter/min flow until the anesthesiologist has advised that there is no evidence of intravascular insufflation. This author prefers to maintain a low flow rate because of the ability of the body to recover from insufflation of $CO_2$ into the vascular system as long as the flow rate is 1 liter/min or less.

Direct puncture with the trocar has been evaluated and has been found to be equally as safe as Veress needle insufflation (37). It has the advantage of more rapid insufflation through a larger trocar site. When direct puncture is performed, it should not be performed in a vertical position, but rather in a position toward the pelvis. Although frequently presented as a safer method of entry than direct puncture, the open Hasson approach has resulted in enterotomies when performed directly over adhesion areas. The open approach carries with it, however, the advantage of avoiding intra-abdominal complications when adhesions do not exist such as laceration of vessels or puncture of the bowel (2).

### Alternate Site for Veress Needle or Direct Puncture

The umbilical site for the initial entry should be avoided if the patient has had a previous operation, either from a Pfannensteil incision or from a vertical incision (39,40). The incidence of bowel adhesions in the region of the umbilicus from these types of procedures can range from 20 to 40% (42). In addition, if a patient has had a previous umbilical hernia repair, the incidence of adhesions to this area is almost 100%. In this case the preferred site of entry is the Palmer site or equivalent right upper quadrant site. The Palmer point (left upper quadrant midclavicular line below the last rib) can be used for either direct puncture or placement of the Veress needle. It is advised by some that the placement of the needle or the trocar be in a 15∞ cephalad direction rather than in a caudad direction (39). In either case, this site is preferable when lower abdominal adhesions are present. By utilizing this site and becoming familiar with it, the laparoscopic gynecological surgeon can avoid trocar injury to bowel and laceration of vessels and adhesions and will find that the visualization, especially with the new three-chip cameras with high light sensitivity and the new 5-mm laparoscopes, is superb.

### Sites for Accessory Trocars

The next step for the laparoscopist to avoid complications is to properly choose the sites for the accessory trocars. All laparoscopists should now be familiar with the position of the inferior epigastric arteries and veins (17,24,30,43). Even in the most obese patients these vessels can usually be visualized because of their close proximity to the peritoneal surface as they cross in near proximity to the round ligament. Avoiding the inferior epigastric vessels is best accomplished for 12-mm trocars by placement of the trocars lateral to these vessels and lateral to the rectus sheath. By placing the trocars in this position, the vasculature of the rectus sheath is also avoided. The position of the circumflex artery and vein in the abdominal wall should also be noted. If the surgeon approaches too closely to the pelvic bone, injuries to both the ileal inguinal and ileal femoral nerves as well as the superficial circumflex artery and vein are possible. A position 2 cm cephalad to the anterior superior spine of ileum and at least 1 to 2 cm lateral to the rectus sheath is usually well suited for placement of trocars. The direction of puncture initially should be straight vertically and then as the point is visualized at the peritoneal surface, the point can be reposititioned, aiming toward the deep pelvis. Smaller trocars, such as 5-mm trocars, can be placed safely medial to the inferior epigastric vessels through the rectus sheath. Regardless of trocar placement, lacerations of vessels in the abdominal wall will occur. All experienced surgeons have encountered this difficulty and written extensively about its ramifications (17,24,30,43). These ramifications include unrecognized lacerations, which are tamponaded until the time of postoperative recovery when a patient becomes hypotensive; episodes wherein bleeding vessels cannot be controlled and laparotomy incisions are required; and episodes where bleeding vessels resulted in severe retroperitoneal hematomas. Laceration of the abdominal wall vasculature should never be ignored as these lacerations can result in morbid consequences. When they occur, the use of an emergency needle technique such as repair using the Carter-Thomason Needle-Point Suture Passer (44) or other equivalent devices such as the Semm emergency needle (31) can promptly control bleeding from these vessels. The use of bipolar energy, while attractive, may not be sufficient to occlude the vessel as episodes of severe hemorrhaging after the attempted control by bipolar cautery of the inferior epigastric vessels may occur.

If abdominal wall vessels are lacerated, both the caudad and cephalad portions should be ligated to ensure complete control.

### Avoid the Use of Insulated Screw Grip Devices

The surgeon should take care to follow the "Rule of Threes" in utilizing electrosurgical instruments through the ports that have been established (45). This rule states that a conductor inside an insulator inside a conductor next to the skin is safe because the energy that is capacitively coupled is dissipated to the skin (for example, unipolar scissors inside its insulating sheath inside a metal conductor trocar sheath next to the skin is safe). Screw grips should be avoided as these can result in much larger portal sizes, sometimes enlarging a 5-mm portal to almost 10 mm and a 10-mm portal to almost 15 mm in size. In addition, they carry the risk of violation of the Rule of Threes, because most screw grips are of insulated material. The surgeon should avoid placing an insulated conducting instrument inside a metal carrier that is in place through an insulated screw grip. Such a situation can occur if a monopolar cautery device is placed through an operating laparoscope, which is passed through a cannula separated from the skin by an insulating screw grip. It can also occur if an operating monopolar instrument is place through a metal irrigation suction cannula, which is placed also through an insulating sleeve (46,47). In these configurations, the possibility of capacitive coupling exists, and injury can occur as a result of discharge of energy from the suction cannula or the operating scope to a piece of bowel in close proximity.

### PATIENT POSITION

The surgeon is prepared now to begin the operation, and the patient can be placed in the Trendelenberg position and the instruments introduced for performance of the intended procedure. A Foley catheter should have been place before initiation of the first incision, draining the bladder well. With all instruments now in place, to minimize complications one must consider the individual procedures to be performed.

### AVOID COMPLICATIONS OF PROCEDURES

### Adhesiolysis

The major dangers of adhesiolysis are 3-fold: 1) loss of control of a vascular portion of the adhesion; 2) injury to an adjacent structure near the adhesion such as bowel; and 3) injury to a structure not visualized because the adhesion obscures the view (28,48,49).

The first principle for adhesiolysis is to have complete visualization of the extent of the adhesion. Therefore, it is of benefit to place the visualizing laparoscope in different positions in the pelvis to obtain a complete view circumferentially of the adhesion (50). In this manner, the possibility of bowel being entrapped into the adhesion is appreciated. In addition, the point of attachment of the adhesion should be carefully visualized to ensure that in fact this is not a hernia formation with bowel being involved. Once these steps have been taken, the adhesion should be placed on traction and treated depending on its level of vascularity and thickness.

#### *Thin adhesions*

Thin adhesions can be treated by sharp dissection with creation of windows and the use of very gently applied bipolar energy for cauterization and scissors dissection (51–53). In addition, bipolar scissors can be utilized for this type of adhesion. Bipolar cutting can be used as well for this type of adhesion. Laser energy can be utilized, but the use of laser-beamed energy such as $CO_2$ should be carefully backstopped because of the possibility of past pointing through thin adhesions (54–56).

#### *Thick adhesions*

Thicker adhesions require traction, the creation of windows, and great care to ensure that the vessels internal to the adhesion are cauterized before cutting. This can be difficult because these adhesions can contain a great deal of fat. Because of this, the use of unipolar energy should be avoided as the fat is not a good carrier of the thermal energy required to control the vessel. The vessel should be isolated to the greatest extent possible and either treated mechanically with clips or carefully cauterized with bipolar cautery (57,58). If large portions of the adhesion as grasped with bipolar cautery, the surgeon must be aware that thermal energy can spread from the heat sink and the fat tissue can become very hot in the process of being cauterized (51). Once control of the vasculature has been obtained, the adhesion can then be cut. Great care must be exercised to place traction on the adhesion such that the traction does not pull on the bowel itself. Cutting should be away from the bowel surface.

### Enterolysis

If adhesions exist between loops of bowel, gynecologists should feel comfortable in calling for general surgical assistance for this delicate type of procedure and should avoid the use of any form of electrical or thermal energy in these regions. Bowel-to-bowel adhesions are best treated by first separation and then observation and limited bipolar cauterization of tendrils of vessels if necessary. If unipolar current is applied in bowel-to-bowel adhesions, remember that the current must pass from the instrument through one of the loops of bowel and can cause thermal damage. Use short bursts of concentrated energy to avoid complications with unipolar energy (52). The blanching of bowel is indicative of deep injury that must be treated by suture control or even excision of the damaged bowel. In general, a carbonized appearance is indicative of a very superficial fulguration energy and may require oversewing and observation. The liberal use of clips is an appropriate approach to treatment of bowel-to-bowel adhesions to avoid risks of other energy sources. Any site of bowel surgery should be carefully investigated and if an opening of bowel is found, a simple technique of repair is to exteriorize that section of the bowel, oversew and repair it, and place it back into the abdominal cavity. Only then return to the original surgery. Liberal use of surgical consultation by gynecologists can avoid serious postoperative complications. Adhesiolysis should be carried out before the initiation of any of the additional procedures planned as the most critical principle of laparoscopic surgery is to maintain a clear complete field of view for the surgery to be performed (28,59–61).

### Adnexal Surgery

To minimize the complications of adnexal surgery, be aware that the most frequent complications involve hemorrhage from the vessels and damage to the ureter. In addition, spill from a possibly malignant tumor of the ovary and spill of a caustic substance from a dermoid cyst can lead to severe complications. To minimize the risk of these complications, the following principles should be applied (62–73).

***Isolate the vascular pedicle***

First, the vascular pedicle of the ovary should be identified and, if possible, a window created to isolate the infundibulopelvic ligament. Then suture ligature can be passed, and an extracorporeal knot applied. An endoloop is then inserted, the vessel is grasped and cut, and the endoloop is placed on the vessel to control the infundibulopelvic ligament with two suture ligatures (40).

The second approach to the infundibulopelvic ligament is to create a window and place the linear stapler through the window across the infundibulopelvic ligament. If this procedure is applied, great care must be taken to ensure that the vessel, when crimped within the linear stapler, does not bunch against the tissue guard, thereby presenting a situation where the staples to be placed through the infundibulopelvic ligament are too few in number to control the vessel. The staple line should be carefully examined after the cut has been applied to ensure that the staple line is hemostatic. Gentle application of bipolar current to the staple line can ensure that no retroperitoneal hematoma forms later. Any staple line or suture line should be examined after removal of pressure and underwater to ensure that no leak or hematoma formation is visualized (74).

A third technique for the infundibulopelvic ligament again involves isolation of the vessel by creation of windows and use of bipolar energy to desiccate/coagulate the vessel. If this technique is used, it is advisable to apply 25 to 30 watts in intermittent bursts. For thicker vessels, it may be necessary to incise into the coagulated area and then reapply the coagulation until the entire vessel has been coagulated and cut. By applying the bipolar energy intermittently, we avoid the problem of carbonization and charring, which results in sticking of the bipolar tines to the tissues and initiating bleeding.

All of these techniques have been used with great success to control the infundibulopelvic ligament. With each of them there have been complications. With application of the staplers there has been the complication of retroperitoneal hemorrhage and bleeding from the incompletely stapled artery. Underwater examination and care with use of bipolar energy behind the staple line in the event of a visualized bleeding area is essential. With the use of bipolar cautery bleeding has occurred as well as injury to the ureter. To avoid this, ensure that the ureter has been identified and place traction on the ovary in an upward direction and somewhat toward the pelvis to ensure that the infundibulopelvic ligament is as far away as possible from the ureter at the point of application of the bipolar energy. In addition, apply short bursts of energy and evaluate for coagulation by visualization during the procedure. The creation of a window also was to avoid damage to the ureter.

### Ovarian Cystectomies

Ovarian cystectomy can be performed safely by laparoscopic procedures. However, it is most important that the contents of the ovarian cyst not be leaked if there is evidence of any possibility of ovarian cancer. In addition, the contents of dermoid cysts can, if spilled, lead to chemical peritonitis and adhesion formation. The recommended procedure is to perform an excision of the cyst while the ovary is placed inside of a pouch- or bag-like structure, and then to remove the cyst intact inside such a bag. While the cyst is in the bag it can be ruptured as long as the fluid is completely contained within the bag. The base of the ovary where the cyst has been removed is frequently best treated by laparoscopic fulguration using spray coagulation or argon beam coagulation. Superficial bipolar energy or deep monopolar energy can be used for bleeding from the ovarian tissue. Suturing is not recommended because of adhesion formation.

### Laparoscopic Burch Procedure

Complications can be avoided during a laparoscopic transperitoneal Burch procedure by ensuring that the initial peritoneal incision is made at least 2 cm above the point of insertion of the bladder into the anterior abdominal wall and by ensuring that the bladder dissection is carried out to as great an extent possible by blunt dissection. Careful dissection of the paravaginal area over the operator's finger will ensure that the bladder is moved well away from the point of insertion of the sutures when they are placed. There is danger of suture placement into the substance of the bladder itself, or if staples are used, they can be inadvertently placed into the bladder. It is important that cystoscopy be performed after laparoscopic bladder suspension to ensure that no evidence of bladder injury exists. Any evidence of blanching on the interior bladder mucosa requires identification of the point of injury by the energy source and long-term drainage for 1 to 3 weeks until the bladder has repaired. In addition, oversewing of the external area of bladder may be required to ensure complete healing. This can be performed laparoscopically.

### Complications of LAVH

During the performance of this procedure the greatest likelihood of damage to the ureter in the ureteric canal exists. The reason for this is the close proximity of the ureter to the uterine artery at the point where the ureter crosses under the uterine artery where there are only 2 cm on average of distance. Any retroperitoneal fibrosis, endometriosis, or scarring can cause traction on the ureter and bring it much closer to the uterine artery than suspected by the surgeon. For this reason, the application of bipolar energy, sutures, or staples in the region of the ureteric canal must be avoided. The ureter must be carefully dissected and identified and all energy sources applied to the uterine artery after careful dissection and identification of the ureter. In spite of efforts to identify the ureter, it has been stapled, cauterized, and sutured. The best way to avoid damage to the ureter is to avoid the ureter entirely, which for most surgeons may involve stopping their dissection above the point of the insertion of the uterine artery into the uterus commonly refered to as the ascending branch of the uterine artery. The use of ureteric stents also assists in identification of the ureter and new developments of lighted stents may make visualizing the ureter easier for the laparoscopic surgeon (6,7,8,9,12,15,25,27,76,78).

### Treatment and Resection of Endometriosis

The most common procedure in which bowel injuries occur is the resection of endometriosis from the rectovaginal septum in the obliterated cul-de-sac (79–81). When one suspects that surgery in this region will be performed, a bowel preparation should be provided preoperatively for the patient. These bowel injuries can be repaired laparoscopically with standard techniques as long as the bowel is cleaned preoperatively. If it is necessary to remove a portion of bowel in a disk excision, this can be removed and repaired. Excision of endometriotic tissue in the region of the uterosacral ligaments and of the rectovaginal septum requires great care and preoperative identification of the ureters. In addition, the placement of both vaginal and rectal probes can help identify planes of dissection. If the ureter cannot be identified and planes of dissection cannot be identified, an open procedure may be of great benefit to both the patient and the surgeon.

### Laparoscopic Tubal Surgery Including Ectopic Pregnancy

Treatment of ectopic pregnancies by laparoscopy has become very routine. The injection of the mesosalpinx with vasopressin solution must be performed carefully as the toxic effects of vasopressin have been documented. A very dilute solution of vasopressin should be utilized in this procedure, ~1 U to 40 ml of normal saline. The most serious complication aside from hemorrhage from the tubal structure itself is retention of products of conception from the laparoscopic procedure. This usually occurs in the proximal section of the tube, and, therefore, careful flushing of the tube, evaluation of the proximal segments, and monitoring of the patient postoperatively with quantitative human chorionic gonadotropin are essential (82).

### EXITING THE ABDOMEN

Once the surgical procedure has been performed, it is necessary to exit the abdomen. Exiting the abdomen is best performed by first removing all gas and evaluating the pelvis after installation of 1 to 2 liters of normal saline or lactated Ringer's solution. Any areas of bleeding should be carefully controlled as these may result in a larger amount of bleeding postoperatively (83–88). All tissue should be evaluated, and the areas should be cleaned carefully. An underwater examination should be carried out in a clear field. If there is any question of potential damage to the bowel, then it should be evaluated by the placement of a sigmoidoscope and the placement of air into the bowel lumen, and evaluation underwater to ensure that there is no gas leakage from the bowel (79). If an operation has been performed close to the bladder or the ureters, the bladder should be evaluated with cystoscopy and the bladder also be fill with 250 ml of methylene blue to evaluate for any leakage areas. If there is any suspicion of damage to the ureter, the integrity of the ureter can be checked by evaluating for the passage of indigo carmine from the ureters at the ureteric orifices while performing cystoscopy. Urological consults should be obtained if there is a question of damage to the ureters during the surgery.

Unrecognized damage to the bowel can be a lethal complication (28). In 66 cases of bowel injury, 13 cases were of large bowel damage and 53 were of small bowel damage. These were cases in which bowel injury occurred but was not recognized at the time of surgery. The bowel can be perforated without obvious leakage of contents. To avoid this risk, severe intra-abdominal adhesions, particularly those involving the small bowel, should only be dealt with when the patient has been adequately prepared and the surgeon has appropriate experience.

The site of any divided adhesions should be carefully inspected. After all laparoscopic surgery, the surgeon should maintain a high index of suspicion of the possibility of bowel perforation. In these 66 cases of unrecognized injury, three patients died, all of whom had a delay in diagnosis of more than 72 hours. Any patient with increasing pain and malaise after laparoscopic surgery should be assumed to have a perforated viscus until proven otherwise (28).

At this point, after complete evaluation of all surgical sites has been performed, the surgeon is prepared to exit the abdomen. This author finds it helpful to place into the peritoneal cavity 20 ml of 0.25% Marcain for relief of pain and to remove all $CO_2$ gas that has been insufflated. In addition, all trocar sites are injected with 0.25% Marcain.

The pain from a bowel injury would remain after the Marcain effect has resolved. This author has found fewer complaints of shoulder pain and incisional pain since using this regimen and less abdominal pain.

The trocar sites of 10 mm and larger instruments should be sutured closed using techniques that allow the closure of both the fascia and the peritoneum (89–108). For this purpose, the Carter-Thomason Fascial Closure Device or an equivalent instrument should be utilized. Herniation into port sites can occur in as many as 3% of 12-mm port sites in lateral positions which are not closed and in 0.3% of those which have been closed by standard techniques that did not include closure of the peritoneum as well as the fascia (26).

All instruments should be removed under direct vision and all sites 10 mm and larger closed under direct vision. The umbilical site can be closed by an alternative method of figure-of-eight 0 Vicryl suture using a UR-6 needle. The skin incisions should be irrigated carefully with an antibiotic solution and then closed in the method preferred by the surgeon. We use a 3-0 nylon which is removed 1 to 2 days after surgery, and the wound closure is reinforced with Steri-Strips.

Postoperatively, the patient's hemoglobin is evaluated in the recovery room to ensure that no vascular compromise has occurred.

### FINAL REMARKS

Remember that the most common laparoscopic complications are generally vascular. The most common catastrophic vascular complications are those that occur at the initiation of the procedure such as placement of a trocar through the aorta. In two cases in which this was reported, the patient survived, but only after the placement of the aortic grafts (83).

The surgeon must also remember that bowel injuries may take from 3 to 7 days to manifest themselves, and return visits of the patients to the surgeon between days 3 and 6 are mandatory to ensure proper follow-up with those patients. Patients should be instructed that any increase in abdominal pain should be evaluated immediately. The pain experienced after laparoscopic surgery should not be assumed to be related to retention of $CO_2$ gas and diaphragmatic irritation until the patient has been personally evaluated by her operating surgeon (28,109,110).

In 127 advanced gynecological laparoscopic procedures reviewed for complications at a community hospital, significant complications were associated with oophorectomy, ovarian cystectomy, myomectomy, pelvic lysis of adhesions, and laparoscopic-assisted vaginal hysterectomy (111). In addition, 100 hysteroscopic procedures were also performed. Uterine perforation occurred in 8% of the hysteroscopic procedures, and there were four postoperative infections in the hysteroscopic group. The patient with the most severe case of hyponatremia had a serum sodium level of 125 mEq/l.

In this study (111), laparoscopic management of ectopic pregnancy resulted in 13% complications. Salpingectomy and paraovarian cystectomy was performed in 11 patients, and there were no complications. Translaparoscopic adnexal surgery, including ovarian cystectomy, oophorectomy, and salpingo-oophorectomy, as performed in 30 patients, and there were complications in 5 patients (17%). The complications included problems with the stapling device, unplanned hospitalization for fever and ileus, tubo-ovarian abscess, difficulty retrieving the ovary, and abdominal wall hematoma (111).

Translaparoscopic myomectomy was performed in 16 patients, (111), with 5 of them having complications (31%). These includes requiring a minilaparotomy to remove a fibroid, requiring an abdominal approach to complete the procedure, and having difficulty with the colpotomy incision, as well as one lost fibroid. LAVH was performed on 15 patients, and there were complications in 9 (60%), including excess blood loss in 8 (mean 767 ml), entering the bladder, and febrile morbidity. Pelviscopic lysis of adhesions was performed on 9 patients, and there was a 55% complication rate (5 patients). Complications included bleeding from a secondary trocar site, excess blood loss, fluid imbalance, transfusion, unplanned hospitalization, general surgical consultation, and an intraoperative intravenous pyleogram. Those who perform a large number of laparoscopic procedures may be surprised at these results, but these are the types of complications that can occur as more and more surgeons begin to approach laparoscopic surgery without, perhaps, proper credentialling or training (111).

During all operative laparoscopic procedures, if lasers operated by beams are used, they should be back stopped. If electrosurgical instruments are used, bipolar electrosurgery is safer, because the current passes only from one conductor plate to another directly in view from the operator. When unipolar cautery is used, the ultimate safety in electrosurgery can be only achieved by active shielding. All instruments should be actively shielded. The active shield leads all capacitively coupled energy back to the surgical unit and detects any energy derived from direct coupling. In the case of direct coupling, the monitor will order the electrosurgical unit to shut down (45).

Remember that the main causes of electrosurgical injuries are inadvertent touching and grasping of tissue during current application, direct coupling between a portion of intestine and a metal probe that is touching the activated probe, insulation breaks in the electrodes, direct sparking to the intestine from the active probe, and current passage to the intestine from recently coagulated, electrically isolated tissue (45).

### REFERENCES

1. Boike GM, et al. Laparoscopically assisted vaginal hysterectomy in the university hospital: Report of 82 cases in comparison with abdominal and vaginal hysterectomy. Am J Obstet Gynecol 1993;6:1690–1701.
2. Hasson HM, Rotman C, Rana N, et al. Experience with laparoscopic hysterectomy. J Am Assoc Gynecol Laparosc 1993;1:1–11.
3. Kadar N. An operative technique for laparoscopic hysterectomy using a retroperitoneal approach. J Am Assoc Gyecol Laparosc 1994;1(4, pt 1):365–377.
4. Langebrekke A, Skar OJ, Urnes A. Laparoscopic hysterectomy: Initial experience. Acta Obstet Gynecol Scand 1992;71:226–229.
5. Liu CY, Reich H. Complications of total laparoscopic hysterectomy in 518 cases. Gynecol Endosc 1994;3:203–208.
6. Liu CY. Laparoscopic hysterectomy. A review of 72 cases. J Reprod Med 1992;37:351–354.
7. Liu CY. Laparoscopic hysterectomy. Report of 215 cases. Gynaecol Endosc 1992;1:73–77.
8. Lyons T. Supracervical laparoscopic hysterectomy: A comparison of morbidity and mortality results with LAVH. J Reprod Med 1993;38:763–767.
9. Maher PJ, Wood EC, Hill DJ, et al. Laparoscopically assisted hysterectomy. Med J Aust 1992;156:316–318.
10. Minelli L, Angeiolillo M, Caione C, et al. Laparoscopically assisted vaginal hysterectomy. Endoscopy 1991;23:64–66.
11. Nezhat CH, Nezhat C, Admon D, et al. Complications of 361 laparoscopic hysterectomies. J Am Assoc Gynecol Laparosc 1994;1(4, pt 2):S25.
12. Nezhat F, Nezhat C, Gordan S, et al. Laparoscopic versus abdominal hysterectomy. J Reprod Med 1992;37:247–250.
13. Padial JG, Sotolongo K, Casey NJ, et al. Laparoscopically assisted vaginal hysterectomy. Report of 75 consecutive cases. J Gynecol Surg 1992;8:81–85.
14. Schwartz RO. Complications of laparoscopic hysterectomy. Obstet Gynecol 1993;81:1022–1024.
15. Carter JE, Bailey TS. Laparoscopic assisted vaginal hysterectomy utilizing the contact-tip Nd:YAG laser. Ann Acad Med Singapore 1994;23:13–17.
16. Carter JE, Ryoo J, Katz A. Laparoscopic assisted vaginal hysterectomy: A case controlled comparative study with total abdominal hysterectomy. J Am Assoc Gynecol Laparosc 1994;1:116–121.
17. Saidi MH, Vancaillie TG, White HA, et al. Complications of advanced operative laparoscopy: A review of 452 cases. J Am Assoc Gynecol Laparosc 1994;1(4, pt 2):S31.
18. Summitt RL, Stovall TT, Lipscomb, et al. Randomized comparison of laparoscopy assisted vaginal hysterectomy to standard vaginal hysterectomy in an outpatient setting. Obstet Gynecol 1992;80:895–901.
19. Woodland, MB. Ureter injury during laparoscopy assisted vaginal hysterectomy with endoscopic linear stapler. Am J Obstet Gynecol 1992;167:756–757.
20. Anonymous. Female sterilization with unipolar electric coagulating devices. MMWR 1981;30:149.
21. Berci G. Complications of laparoscopic surgery. Surg Endosc 1994;8:165–166.
22. Garry R. The Achilles heel of minimal access surgery. Gynaecol Endosc 1994;3:201–202.
23. Hogdall C, Roosen JU. Incarcerated hernia following laparoscopy. Acta Obstet Gynecol Scand 1987;66:735–736.
24. Holtz G. Laparoscopy in the massively obese female. Obstet Gynecol 1987;69:423–434.
25. Kadar N, Lemmerling L. Urinary tract injuries during laparoscopically assisted hysterectomy: Causes and prevention. Am J Obstet Gynecol 1993;170(1, pt 1):47–48.
26. Kadar N, Reich H, Liu CY, et al. Incisional hernias after major laparoscopic gynecologic procedures. Am J Obstet Gynecol 1993;168:1493–1451.
27. Khare VK, Martindent DC, Kai GHJ. Buttonhole ulceration and perforation of the rectum. J Am Assoc Gynecol Laparosc 1993;1:12–15.
28. Soderstrom RM. Bowel injury litigation after laparoscopy. J Am Assoc Gynecol Laparosc 1993;1:74–77.
29. Baagsgaard SE, Bille S, Egeblad K. Major vascular injury during gynecological laparoscopy. Report of a case and review of published cases. Acta Obstet Gynecol Scand 1989;68:283–295.
30. Berqavist D, Berqavist A. Vascular injuries during gynecologic surgery. Acta Obstet Gynecol Scand 1987;66:19–23.
31. Semm K. Endoscopic intra-abdominal surgery. Keil: Christian-Albrects-Universitat, 1984:4.
32. Azziz R, Steinkamph MP, Murphy A. Postoperative recuperation: Relation to the extent of endoscopic surgery. Fertil Steril 1989;51:1061–1067.
33. Friedman RL, Friedman IH, McSherry CK. Pneumothorax associated with laparoscopic cholecystectomy. Surg Endosc 1994;8:797–799.
34. Pasulka PS, Bristrian BR, Benotti PN, et al. The risks of surgery in obese patients. Ann Intern Med 1986;104:540–546.
35. Batres F, Barclay DL. Sciatic nerve injury during gynecologic procedures using low lithotomy position. Obstet Gynecol 1983;62(suppl):92S–94S.

36. Dabirashrafi H, Mohammad K, Tabrizi MM, et al. The use of Veress' needle and 10 mm trocar vs. direct trocar insertion in the beginning of laparoscopy. J Am Assoc Gynecol Laparosc 1994;1(4, pt 2):S9.
37. Nezhat FR, Silfen SO, Evans D, et al. Comparison of direct insertion of disposable and standard reusable laparoscopic trocars to pneumoperitoneum with Veress' needle. Obstet Gynecol 1991;78:148–150.
38. Perone N. Laparoscopy using a simplified open technique: A review of 585 cases. J Reprod Med 1992;39:921–924.
39. Chang FH, Lee CL, Soong YK. Use of Palmer's point for insertion of the operative laparoscope in patients with severe pelvic adhesions: Experience with 17 cases. J Am Assoc Gynecol Laparosc 1994;1(4, pt 2):S7.
40. Childers JP. Gynecol Oncol 1993;50:221.
41. Levrant SG, Bieber E, Barnes R. Risks of anterior abdominal wall adhesions increase with number and type of previous laparotomy. J Am Assoc Gynecol Laparosc 1994;1(4, pt 2):S19.
42. Bieber EJ, Levrant S. The risk of anterior abdominal wall adhesions in patients with previous umbilical hernia repair. J Am Assoc Gynecol Laparosc 1994;1(4, pt 2):S4.
43. Vasquez JM, Demarque AM, Diamond MP. Vascular complications of laparoscopic surgery. J Am Assoc Gynecol Laparosc 1994;1:163–167.
44. Carter JE. A new technique of fascial closure for laparoscopic incisions. J Laparoendosc Surg 1994;4:143–147.
45. VanCallie TG. Electrosurgery at laparoscopy: Guidelines to avoid complications. Gynaecol Endosc 1994;3:143–150.
46. Voyles CR, Tucker RD. Unrecognized hazards of surgical electrodes passing through metal suction irrigation devices. Surg Endosc 1994;8:185–187.
47. Nduka CC, Super PA, Monson JR, et al. Cause and prevention of electrosurgical injuries in laparoscopy. J Am Coll Surg 1994;179:161–170.
48. Francois Y, Moure TP, Tomauglu K, et al. Postoperative adhesive peritoneal disease: Laparoscopic treatment. Surg Endosc 1994;8:781–783.
49. Levy BS, Soderstrom RM, Dail DH. Bowel injuries during laparoscopy: Gross anatomy and histology. J Reprod Med 1985;30:168–179.
50. Sackier G. Maintaining a clear view in laparoscopic surgery. Surg Endosc 1994;8:824–825.
51. Phipps, JH. Thermometry studies with bipolar diathermy during hysterectomy. Gynaecol Endosc 1994;3:35–37.
53. Rioux JE, Cloutier D. A new bipolar instrument for laparoscopic double sterilization. Am J Obstet Gynecol. 1974;119:737.
54. Bohn B, Milson JW, Kitago K, et al. Monopolar electro surgery and Nd:YAG contact laser in laparoscopic intestinal surgery. Surg Endosc 1994;8:677–681.
55. Gunyk YS, Keller FS, Halpern NB. Hepatic artery pseudoaneurysm and hemobilia following laser laparoscopic cholecystectomy. Surg Endosc 1994;8:201–204.
56. Hunter JG. Laser or electrocautery for laparoscopic cholecystectomy? Am J Surg 1991;161:345–349.
57. Moore JP, Silvas SE, Vennes JA. The evaluation of bipolar electrocoagulation in canine stomachs. Gastrointest Endosc 1978;24:148–151.
58. Tucker RD, et al. A comparison of neurologic application of bipolar versus monopolar: Five French electrosurgical probes. J Urol 1989;141:662–665.
59. Nezhat C, Nezhat F, Ambroze W, et al. Laparoscopic repair of small bowel and colon. Surg Endosc 1993;7:88–89.
60. Thompson BH, Wheeless CR. Gastrointestinal complications of laparoscopic sterilization. Obstet Gynecol 1973;41:669.
61. Berry SM, Ose KJ, Bell RH, et al. Thermal injury of the posterior duodenum during laparoscopic colpocystectomy. Surg Endosc 1994;8:197–200.
62. Canis M, Mage G, Poul JL, et al. Laparoscopic management of suspicious adnexal masses. J Am Assoc Gynecol Laparosc 1994;1(4, pt 2):S31.
63. Luxman D, Cohen Jr, Avni A, et al. Laparoscopic conservative cystectomy of ovarian benign cystic teratoma. J Am Assoc Gynecol Laparosc 1994;1(4, pt 2):S19.
64. Marana R, Vittori G, Campo S, et al. Operative laparoscopy for adnexal cystic masses in patients under 40 years of age. J Am Assoc Gynecol Laparosc 1994;1(4, pt 2):S20.
65. Papasakelariou C, Saunders D, Delarosa A. Comparative study of laparoscopic oophorectomy. J Am Assoc Gynecol Laparosc 1994;1(4, pt 2):S27.
66. Saidi MH, Sarosdy MF, Hollimon PW. Intestinal obstruction and bilateral ureteral injuries after laparoscopic oophorerectomy in a patient with severe endometriosis and a large endometrioma. J Am Assoc Gynecol Laparosc 1994;1(4, pt 2):S31.
67. Saks M, Deckardt T. Laparoscopic treatment of benign ovarian dermoid cyst. J Am Assoc Gynecol Laparosc 1994;1(4, pt 2):S31.
68. Shwadyer JM. Use of transvaginal sonography prior to operative endoscopy. J Am Assoc Gynecol Laparosc 1994;1(4, pt 2):S33.
69. Slangen T, Beretta P, Catalano G, et al. Bag surgery as part of a protocol to treat ovarian masses by laparoscopy. J Am Assoc Gynecol Laparosc 1994;1(4, pt 2):S34.
70. Tewilde RL, Hesseling M. Safety and efficacy of the endosurgical management of ovarian cysts in premenopausal women: A prospective study. Gynaecol Endosc 1994;3:101–104.
71. Ulrich U, Keckstein J, Karageorgieda E. Ovarian mature teratoma: Technical aspects of laparoscopic removal. Gynaecol Endosc 1994;3:169–172.
72. Vittori G, Rosetti A, Marana R, et al. Operative laparoscopy for the treatment of ovarian dermoids. J Am Assoc Gynecol Laparosc 1994;1(4, pt 2):S39.
73. Parker WH, Levine RL, Howard FM, et al. Management of selected adnexal masses in postmenopausal women by operative laparoscopy. A multicentral study. J Am Assoc Gynecol Laparosc 1994;1(4, pt 2):S27.
74. Nezhat C, Nezhat F, Bess O, et al. Injuries associated with the use of a linear stapler during operative laparoscopy: A review. J Gynecol Surg 1993;9:144–150.
75. Carter JE. Laparoscopic Burch procedure for stress urinary incontinence—The Carter modification. Proceedings of the Second European Congress of Gynecologic Endoscopy and New Surgical Techniques, Heidelberg, Germany, October 21–23, 1993. Editors Edg. Bastert: Monduzzi Editore, 1994;291–293.
76. Baumann H, Jaeger P, Huch A. Ureteral injuries after laparoscopic tubal sterilization by bipolar electrocoagulation. Obstet Gynecol 1988;71:483–485.
77. Dicker RC, Greenspan JR, Strauss LT, et al. Complications of the abdominal and vaginal hysterectomy among women of reproductive age in the United States. Am J Obstet Gynecol 1982;144:841–848.
78. Gambone JC, Raiter RC, Lench JB. Quality assurance indicators and short-term outcome of hysterectomy. Obstet Gynecol 1990;76:831–845.
79. Nezhat C, Nezhat F, Pennington E, et al. Laparoscopic excision and primary repair of the anterior rectal wall for the treatment of full thickness endometriosis. Surg Endosc 1994;8:682–685.
80. Sutton C. The role of laparoscopic surgery and the treatment of minimal to moderate endometriosis. Gynaecol Endosc 1993;2:131–133.
81. Carter JE. Laparoscopic treatment of chronic pelvic pain in 100 adult women. J Am Assoc Gynecol Laparosc 1995;2:255–262.
82. De Waat MJ, De Blok S, Hemrika DJ. Complications of laparoscopic treatment of tubal ectopic pregnancies. Gynaecol Endosc 1994;3:173–175.
83. Apelgren KN, Scheeres DE. Aortic injury: A catastrophic complication of laparoscopic colpocystectomy. Surg Endosc 1994;8:689–691.
84. Nezhat F, Brill A, Nezhat C, et al. Traumatic hypogastric artery bleeding: Control with bipolar desiccation during operative laparoscopy. J Am Assoc Gynecol Laparosc 1994;1:171–173.
85. Ponsky JL. Complications of laparoscopic cholecystectomy. Am J Surg 1991;161:393–395.

86. Selvanov V, Chi HS, Alverdy JC, et al. Mortality and retroperitoneal hematoma. J Trauma 1984;24:1022–1026.
87. Strasberg SM, Samabria JR, Clavien PA. Complications of laparoscopic cholesystectomy. Can J Surg 1992;35:275–280.
88. Stuttmann R, Skelly A. Laparoscopy and its complications. Minimally Invas Ther 1994;3(suppl 2):25–30.
89. Ballem RV, Kenny R, Giuliano M. Small bowel obstruction following laser laparoscopic cholecystectomy: A case study. J Laparoendosc Surg 1993;3:313–314.
90. Bendavid R. Incisional parapubic hernias. Surgery 1990;108:898–901.
91. Bourke JB. Small intestinal obstruction from a Richter's hernia at the site of insertion of a laparoscope. Br Med J 1977;2:1393.
92. Bucknall TE, Cox PJ, Ellis H. Burst abdomen and incisional hernia: A prospective study of 1,129 major laparotomies. Br Med J 1982;284:931–933.
93. Kiely EM, Spitz L. Layered versus mass closure of abdominal wounds in infants and children. Br J Surg 1985;72:739–740.
94. Kiilholma P, Makinen J. Incarcerated Richter's hernia after laparoscopy: A case report. Eur J Obstet Gynecol Reprod Biol 1988;28:75–77.
95. Ellis H, Gajraj H, George CD. Incisional hernias: When do they occur? Br J Surg 1983;70:290–291.
96. Flowers SS, Fenoglio ME, Williams M. Richter's hernia: A rare complication of laparoscopy. Presented at the Society of Laparoendoscopic Surgeons Annual Meeting, December 1993.
97. George JP. Presentation and management of laparoscopic incisional hernias. J Am Assoc Gynecol Laparosc 1994;1(4, pt 2):S12.
98. Hass BE, Schrager RE. Small bowel obstruction due to Richter's hernia after laparoscopic procedures. J Laparoendosc Surg 1993;3:421–423.
99. Maio A, Ruchman RB. CT diagnosis of post laparoscopic hernia. J Comput Assist Tomog 199x;15:1054–1055.
100. McMurrick PJ, Polglase AL. Early incisional hernia after use of the 12 mm port for laparoscopic surgery. Aust N/Z J Surg 1993;63:574–575.
101. Montz FJ, Holschneider CH, Munro M. Incisional hernia following laparoscopy: Survey of the American Association of Gynecologic Laparoscopists. J Am Assoc Gynecol Laparosc 1994;1(4, pt 2):S23.
102. Patterson M, et al. Postoperative bowel obstruction following laparoscopic surgery. Am Surg 1993;59:656–657.
103. Sauer M, Jarrett JC. Small bowel obstruction following diagnostic laparoscopy. Fertil Steril 1984;42:653–654.
104. Schiff I, Naftolin F. Small bowel incarceration after uncomplicated laparoscopy. Obstet Gynecol 1974;43:674–675.
105. Silva AL, Petroianu A, Horizonte B. Incisional hernias: Factors influencing development. South Med J 1991;84:1500–1504.
106. Thomas AG, McLymont F, Moshipur J. Incarcerated hernia after laparoscopic sterilization: A case report. J Reprod Med 1990;35:639–640.
107. Toub DB, Campion MJ. Herniation through a 5 mm laparoscope trocar site: A case report. J Am Assoc Gynecol Laparosc 1994;1(4, pt 2):S27.
108. Tsui S, Ellis H. Healing of abdominal incisional hernia in infant rats. Br J Surg 1991;78:927–929.
109. Toub DB, Zubernis J, Campion MJ, et al. Resolution of free intraperitoneal air of laparoscopy: Utility of the abdominal radiography in the diagnosis of bowel injury. J Am Assoc Gynecol Laparosc 1994;1(4, pt 2):S37.
110. Redwine DB. Symptomatic internal hernia of the broad ligament: A complication of electrocoagulation therapy of endometriosis. Obstet Gynecol 1989;73:495–496.
111. Smith DC, Donohue LR, Waszak SJ. A hospital review of advanced gynecologic endoscopic procedures. Am J Obstet Gynecol 1994;170:1635–1642.

